# Hidradenocarcinoma of the Abdominal Wall Treated With Wide Surgical Excision and Adjuvant Radiotherapy

**DOI:** 10.7759/cureus.14724

**Published:** 2021-04-27

**Authors:** Rafey Rehman, Bryan Squires, Muhammad Osto, Thomas Quinn, Peyman Kabolizadeh

**Affiliations:** 1 Department of Radiation Oncology, Beaumont Health, Royal Oak, USA; 2 Department of Dermatology, Wayne State University School of Medicine, Detroit, USA

**Keywords:** hidradenocarcinoma, abdomen, radiation oncology, oncology, tumor

## Abstract

Hidradenocarcinomas are rare malignant sweat gland tumors that typically arise in the head and neck area. To the best of our knowledge, this is the only reported instance of hidradenocarcinoma of the abdominal wall as well as the first case arising from a region of prior trauma. A 72-year-old female presented with a left abdominal wall lesion, which she had first noticed after an injury to the area. Initially, the lesion remained stable in size, after which it became mildly pruritic, progressive in size, and expressive of a clear, non-odorous discharge. Imaging demonstrated a heterogeneous cystic density. Surgical pathology revealed a malignant dermal adnexal neoplasm composed of pleomorphic polygonal cells and focal intracytoplasmic lumina lined by eosinophilic cuticles, as well as areas of ductal differentiation, apocrine differentiation, and mucinous metaplasia. Surgical excision of the mass was performed, followed by adjuvant external beam radiotherapy (EBRT). The patient had no long-term toxicities or clinical evidence of local disease recurrence as of one year post-surgery and six months post-EBRT. Early diagnosis and treatment are essential to improving outcomes in patients with hidradenocarcinomas. Frequent follow-up is equally important, as these tumors have high recurrence rates.

## Introduction

Hidradenocarcinomas are rare sweat gland malignancies, accounting for less than 0.0001% of tumors reported [1-3]. Based on immunohistochemical differences, hidradenocarcinomas may be reported with varying names, such as malignant nodular hidradenoma, malignant clear cell hidradenoma, malignant clear cell eccrine carcinoma, malignant clear cell acrospiroma, or primary mucoepidermoid cutaneous carcinoma [4-6]. These tumors typically arise in the head and neck area - usually the face - and present with non-specific clinical signs and symptoms, such as a solitary cutaneous or subcutaneous lump [7]. We hereby report an unusual case of hidradenocarcinoma of the abdominal wall, along with a discussion of the differential diagnosis and a brief review of the literature.

## Case presentation

A 72-year-old female with a past medical history of hypertension, gastroesophageal reflux disease, and degenerative disc disease presented with a left abdominal wall lesion, which she had first noticed arising in an area of previous trauma from a motor vehicle collision three years prior to symptom onset. Initially, the lesion was described as asymptomatic with a cystic appearance and had remained stable in size and appearance for a prolonged period. However, three months prior to presentation, the lesion became mildly pruritic, progressive in size, and expressive of a clear, odorless discharge. The patient was referred to a general surgeon, at which point she was noted to have a left abdominal wall subcutaneous mass.

**Figure 1:**
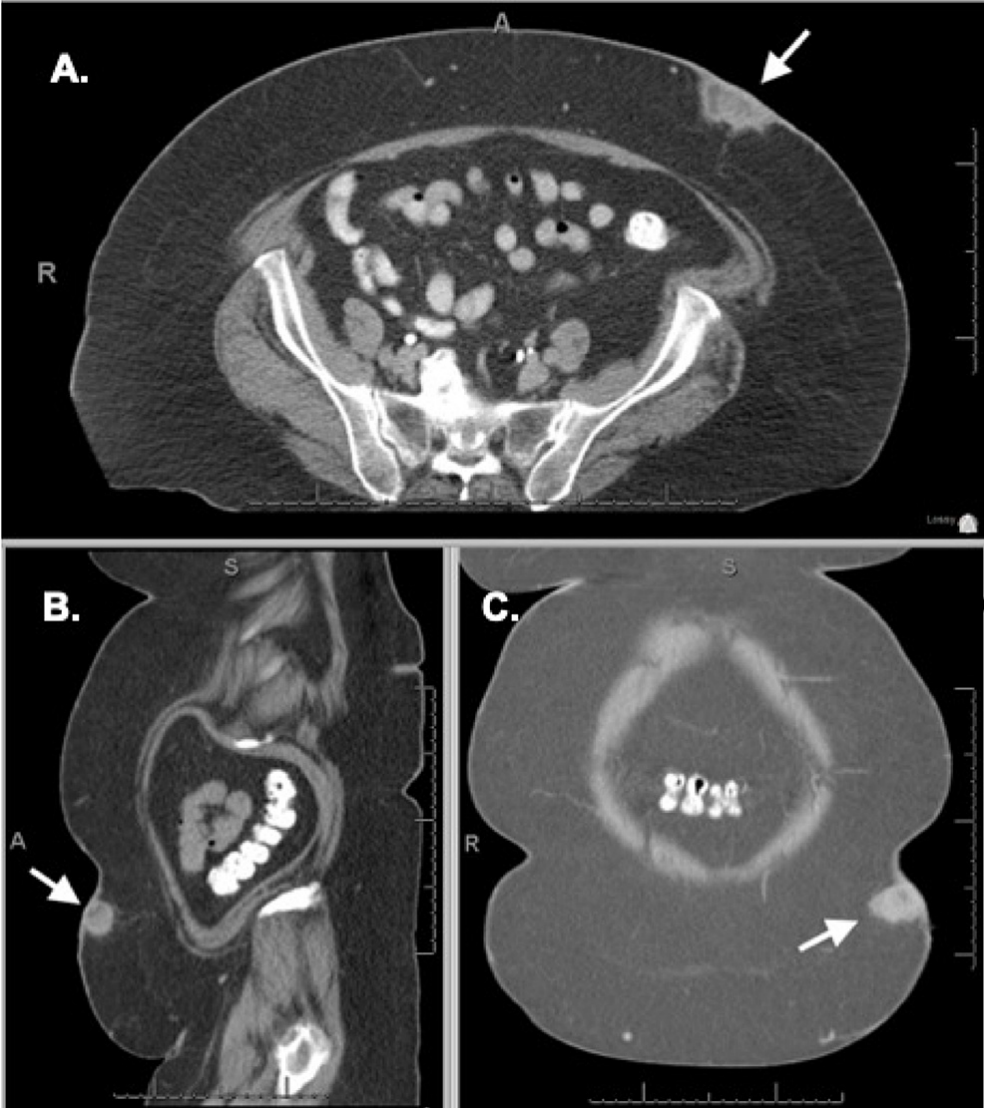
Computed tomography of the abdomen and pelvis with intravenous contrast Selected slices from the patient’s initial computed tomography scan including axial (A), sagittal (B), and coronal (C) views. Seen in the left superficial abdominal wall is a hyperintense lesion corresponding to the patient’s hidradenocarcinoma (indicated by the white arrow).

Computed tomography of the abdomen and pelvis with intravenous contrast was performed, which revealed a heterogeneous cystic density in the left abdominal subcutaneous tissues measuring 4.7 x 2.5 x 2.8 cm with internal fat density, skin thickening, and retraction (Figures 1A-1C). The study was otherwise unremarkable with no significant mesenteric or retroperitoneal adenopathy. No prior imaging studies were available for comparison.

The patient underwent complete excision of the lesion. Upon pathological assessment, the specimen was grossly composed of a 7.5 x 4.5 cm ellipse excised to a depth of 4.7 cm, which contained a 2.6 x 2.7 x 2.0 cm firm mass with solid white components, as well as focal areas of hemorrhage, necrosis, and cavitation with a brown and bright yellow periphery. Microscopically, sections were composed of a malignant dermal adnexal neoplasm composed of pleomorphic polygonal cells with prominent nuclei, abundant clear to eosinophilic cytoplasms, and frequent mitoses. Focal intracytoplasmic lumina lined by eosinophilic cuticles were noted, as were areas of ductal differentiation, apocrine differentiation, and mucinous metaplasia. There was variable cystic degeneration and central necrosis. The surrounding stroma was sclerotic, exhibiting cancerization of sweat ducts and containing surrounding granulomatous response consistent with tumor rupture. On immunohistochemical stains, tumor cells were diffusely positive for epithelial membrane antigen (EMA), p63, and cytokeratin 7 (CK7), with patchy positivity noted for gross cystic disease fluid protein 15 (GCDFP-15), as well as focal expression of mammaglobin. Tumor cells were negative for expression of CD68, S100, CD34, cytokeratin 20 (CK20), and estrogen receptor (ER). These findings were consistent with a diagnosis of hidradenocarcinoma with apocrine differentiation. Surgical margins were clear but close (<0.1 cm) and there was no evidence of lymphovascular invasion.

Three weeks following surgery, the patient underwent computed tomography of the chest, which revealed bilateral linear scarring versus atelectasis at the costophrenic angles with a 2-mm pulmonary nodule in the right upper lobe with scattered mediastinal lymph nodes in the prevascular and precarinal regions, which were <1 cm in short axis, not enlarged by size criteria. The study was otherwise unremarkable. The patient was discussed in a multidisciplinary conference, at which point positron emission tomography (PET) for completion of staging was recommended. F18-fluorodeoxyglucose PET was performed three months postoperatively and revealed the nonspecific mediastinal lymph nodes to have a maximum standardized uptake value (SUV) of 2.74, considered to be more likely reactive. The study was otherwise unremarkable.

**Figure 2:**
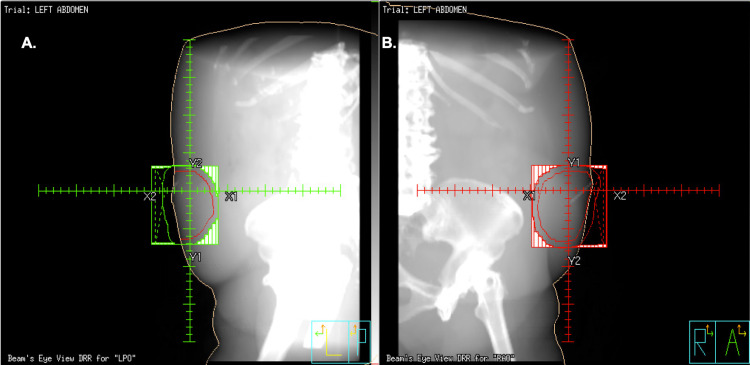
Digitally reconstructed radiograph (DRR) images Digitally reconstructed radiograph (DRR) images showing the beam’s eye view of the left posterior oblique (LPO; A) and right anterior oblique (RPO; B) fields, shaped with multi-leaf collimators around the planning target volume (marked with a red contour along the skin surface). Wedges were utilized to improve homogeneity. Daily tissue-equivalent bolus applications were performed to ensure prescription dose at surface.

**Figure 3:**
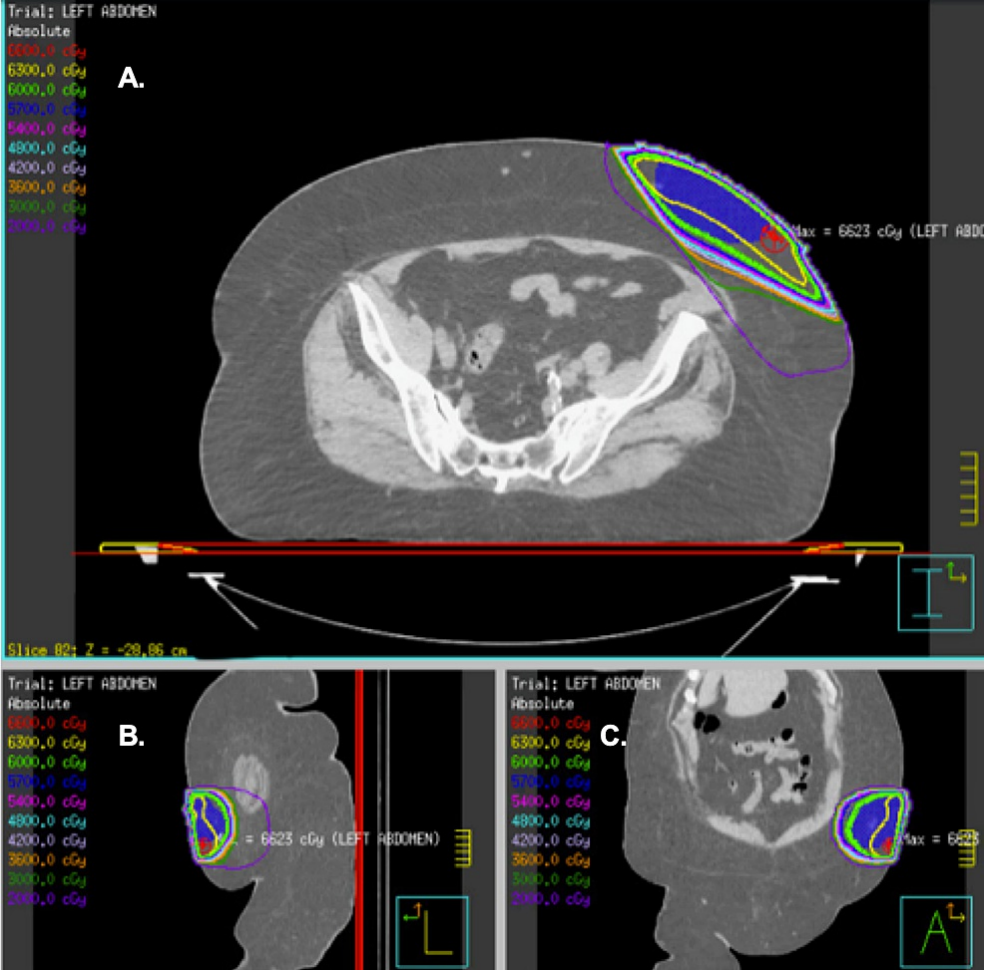
Isodose lines (IDLs) from the treatment plan IDLs from the patient’s treatment plan are displayed on axial (A), sagittal (B), and coronal (C) computed tomography simulation slices. The thick green line represents the prescription IDL (6000 cGy, 100% isodose line); the other IDLs are indicated with the color key in the top-left of each image. The blue color-washed volume indicates the planning target volume (PTV).

Given the aggressive histology and close margins, adjuvant external beam radiotherapy (EBRT) was recommended. She initiated EBRT 20 weeks postoperatively, with 60 Gy delivered in 30 fractions via a 3D conformal wedge-pair arrangement of right anterior oblique and left posterior oblique 10 MV photon beams with daily application of a 5-mm tissue-equivalent bolus (Figures 2A, 2B, 3A-3C). She was noted to develop mild skin erythema in the treatment field after approximately 22 fractions, which progressed to Common Terminology Criteria for Adverse Events (CTCAE) Version 4.0 grade 3 moist skin desquamation by the end of treatment, for which aluminum acetate soaks were prescribed. In addition, she did note mild fatigue during treatment. Otherwise, treatment was well tolerated.

The patient was lost to follow-up for an extended period, but was contacted by telephone approximately 20 months following completion of EBRT, at which point she noted the skin of her abdomen had completely healed and had no complaints of palpable local recurrence or radiation toxicity.

## Discussion

Hidradenocarcinomas are extremely rare sweat gland tumors that classically present as asymptomatic cutaneous lesions [8]. They are usually diagnosed during the fifth to seventh decade of life with a slight predilection for females but without a racial preference [4,9]. The tumor typically presents in the head and neck region and occasionally appears in the extremities [4,10]. There have been rare presentations of hidradenocarcinoma in the groin, scalp, or abdomen [3]. To our knowledge, this is the first reported instance of hidradenocarcinoma of the abdominal wall. Interestingly, the patient’s presentation involved a symptomatic lesion several years following physical trauma from a motor vehicle collision, though it is unclear whether this event contributed to the development of the malignancy or simply brought attention to the lesion.

Though hidradenocarcinomas may arise from an existing benign hidradenoma, they are much more likely to arise de novo [2,5]. Upon gross pathologic examination, the tumor classically presents as well-circumscribed nodules in the superficial skin [8]. Microscopically, the tumor consists of two primary cell types: eosinophilic spindle cells and larger clear cells [8]. The spindle cells typically reside in the periphery of the tumor, while the clear cells are concentrated with glycogen, allowing them to be observed with the periodic acid-Schiff stain [11]. To histologically differentiate between hidradenocarcinomas and hidradenomas, one can look for characteristics such as a lack of demarcation, an increased mitotic rate of clear cells, pleomorphic cells, and invasion into adjacent tissue [3,12]. Similar to other eccrine tumors, hidradenocarcinomas express markers such as EMA, carcinoembryonic antigen (CEA), cytokeratin, and S100. In this case, EMA and cytokeratin positivity was noted, though S100 expression was negative. Various immunohistochemical expression patterns have been observed, such as positivity for androgen receptor (AR), ER, epidermal growth factor receptor (EGFR), progesterone receptor (PR), and human epidermal growth factor receptor (HER-2), with rates of 36%, 27%, 85%, 16%, and 12%, respectively [13]. In this case, the tumor stained negative for ER.

Growth of hidradenocarcinomas is variable, ranging from months to years before taking a more aggressive course including rapid enlargement and regional lymph node metastasis [11]. Despite this progression, most patients remain asymptomatic even after metastasis occurs [8]. In this case, the initial lesion was noticed three years prior to presentation and was relatively dormant until three months prior to presentation, at which point rapid growth occurred without clinical evidence of metastasis.

The optimal treatment for hidradenocarcinoma is unclear due to its rarity and conflicting reports of the effectiveness of adjuvant treatment [10]. Contemporary data suggest that wide surgical excision is indicated [8]. Though not recommended routinely, adjuvant radiotherapy may also be used in patients where surgery is not possible or if the tumor has aggressive features increasing the risk of recurrences, such as inadequate surgical margins, anaplastic histology, dermal invasion, or perineural invasion [14,15]. Various chemotherapy strategies have been proposed, such as the use of regimens involving 5-fluorouracil (5FU) or its oral prodrug, capecitabine. Recommended second-line agents include doxorubicin, platinums, cyclophosphamide, bleomycin, or vincristine [10,14]. Given the prevalence of AR, ER, and HER-2 expression, hormonal agents and trastuzumab have also been used as a targeted therapy to stabilize the solid tumor [10]. However, the efficacy of chemotherapy in the treatment of sweat gland tumors is unclear and requires further investigation [16]. In this case, adjuvant EBRT was offered in an attempt to reduce the risk of recurrence felt to be conferred by the close surgical margins and aggressive histology [17].

Given its poor prognosis, with five-year survival rates after surgery in the range of 30%, it is possible that an earlier diagnosis of hidradenocarcinoma may improve outcomes by allowing for adequate resection, appropriate adjuvant therapies, and prevention of metastatic disease [8]. With rates of recurrence ranging from 10% to 50%, these patients should be followed monthly with attention to possible spread to local lymph nodes or adjacent organs [10,11]. It should be noted that there have been reported cases of long-term survivors without recurrence. Labbardi et al. described a case of hidradenocarcinoma of the heel with metastasis to the inguinal lymph nodes treated with wide local excision of the tumor, inguinal lymphadenectomy, and subsequent radiotherapy, with no evidence of recurrence even after 34 months of follow up [18]. In addition, Khan et al. described a case of hidradenocarcinoma of the scalp treated with wide surgical excision and cisplatin chemotherapy concomitant with radiotherapy, after which the patient was free of recurrence after a five-year follow-up period [19]. Further studies must be conducted to determine the optimal treatment strategy for hidradenocarcinoma.

## Conclusions

Hidradenocarcinomas are rare sweat gland malignancies that typically arise in the head and neck region. Given the rarity of these tumors and conflicting reports in the literature, the optimal treatment for hidradenocarcinomas is unclear. In our patient, the hidradenocarcinoma occurred in the abdominal wall and was managed using surgical excision and EBRT. Although patient was lost to follow-up, a subsequent phone visit revealed complete healing of her abdominal skin without any recurrences or complications of radiation therapy. Early diagnosis and treatment is essential to improving outcomes in patients with hidradenocarcinomas. Frequent follow-up is equally important, as these tumors have high recurrence rates. Further studies must be conducted to determine the optimal treatment strategy for hidradenocarcinoma.
